# A Review of the Immunomodulating Components of Maternal Breast Milk and Protection Against Necrotizing Enterocolitis

**DOI:** 10.3390/nu12010014

**Published:** 2019-12-19

**Authors:** Lila S. Nolan, Olivia B. Parks, Misty Good

**Affiliations:** 1 Department of Pediatrics, Division of Newborn Medicine, Washington University School of Medicine, St. Louis, MO 63110, USA; lilanolan@wustl.edu; 2 University of Pittsburgh School of Medicine, Medical Scientist Training Program, Pittsburgh, PA 15213, USA; Parks.Olivia@medstudent.pitt.edu

**Keywords:** breast milk, necrotizing enterocolitis, prematurity, immunity, newborn, inflammation

## Abstract

Breast milk contains immunomodulating components that are beneficial to newborns during maturation of their immune system. Human breast milk composition is influenced by an infant’s gestational and chronological age, lactation stage, and the mother and infant’s health status. Major immunologic components in human milk, such as secretory immunoglobulin A (IgA) and growth factors, have a known role in regulating gut barrier integrity and microbial colonization, which therefore protect against the development of a life-threatening gastrointestinal illness affecting newborn infants called necrotizing enterocolitis (NEC). Breast milk is a known protective factor in the prevention of NEC when compared with feeding with commercial formula. Breast milk supplements infants with human milk oligosaccharides, leukocytes, cytokines, nitric oxide, and growth factors that attenuate inflammatory responses and provide immunological defenses to reduce the incidence of NEC. This article aims to review the variety of immunomodulating components in breast milk that protect the infant from the development of NEC.

## 1. Introduction

Necrotizing enterocolitis (NEC) is a severe gastrointestinal disease in preterm infants with associated mortality as high as 50% in cases that require surgical intervention [1]. NEC occurs in 1–5% of patients admitted to a neonatal intensive care unit (NICU), and increased incidence and fatality occurs in infants with prematurity and low birth weight [2]. The etiology of NEC is complex, and pathogenesis is attributed to inflammation of the neonatal gastrointestinal tract by triggers such as commercial formula feeds, intestinal dysbiosis, and immaturity of gut mucosal immunity. Treatment of NEC requires withholding enteral feeds and potent antimicrobial agents, and these infants are at risk of adverse long term outcomes. Identification of factors that contribute to the prevention of NEC remains a high priority in neonatal research. There is a consensus regarding the protective nature of breast milk in the prevention of NEC development. Human breast milk, in contrast to commercial formulas, contains soluble and cellular components that provide infants with passive immunity to their gastrointestinal tract. These antimicrobial and bioactive factors are multi-functional and anti-inflammatory, with an established protective role against the development of NEC. An early prospective study of 926 infants showed that exclusively formula-fed preterm infants were six to ten times more likely to acquire NEC as compared to preterm infants nourished with human milk alone [3]. Furthermore, an analysis of 243 infants in a randomized trial showed that preterm infants less than 30 weeks’ gestation who received maternal milk had reduced incidence of late-onset sepsis or NEC as compared with preterm infants who received donor breast milk or commercial formula [4]. Sullivan and colleagues demonstrated in a randomized, controlled, multicenter trial that extremely premature infants who received only human milk, including human milk-derived fortification, had decreased rates of NEC compared to those infants exposed to bovine milk-derived products with a number needed to treat of 10 infants to prevent one NEC case [5].

**Figure 1:**
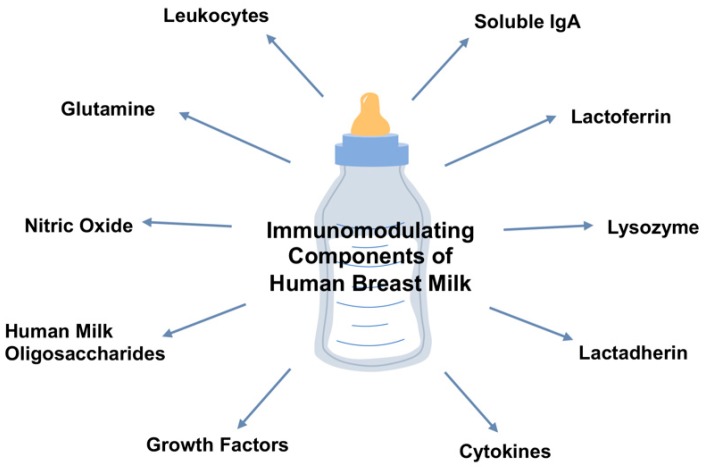
Overview of the immunomodulatory components of maternal breast milk.

Breast milk composition is complex, dynamic, and influenced by a variety of maternal factors. Immunoglobulins, antimicrobial peptides, growth factors, human milk oligosaccharides, cytokines, L-glutamine, and nitric oxide in breast milk maintain roles in the enhancement of the neonatal intestinal barrier function and the reduction of NEC. This article aims to review the protective role of breast milk and its components against NEC, as shown in Figure 1.

## 2. Breast Milk and the Host-Microbial Relationship

### 2.1. Maternal Soluble IgA

Bioactive components of maternal milk, particularly immunoglobulin A (IgA), have known influential effects on the neonatal microbiota. IgA is the most plentiful antibody in human milk and comprises a significant portion of total protein content in colostrum [6]. IgA produced by the maternal mammary glands undergoes proteolytic cleavage to release secretory IgA (sIgA), permitting transport into human milk [7]. Breast milk sIgA provides critical antimicrobial defense to the neonatal gastrointestinal tract through inhibition of pathogen attachment to mucosal surfaces, neutralization of microbial toxins, and provision of passive immunity. IgM and IgG are of lesser abundance in human milk but also have known immune-surveillance properties.

The sIgA levels in breast milk decrease over time during the postpartum period [8]. Recent studies have identified no difference in breast milk sIgA concentration of preterm versus term breast milk [6,8], although Mehta and Petrova identified that preterm milk contains a higher concentration of sIgA in the first six to eight days of lactation [9]. Breast milk sIgA is a critical component, as it promotes colonization of commensal microbiota, decreasing the activity of pattern recognition receptors and subsequent downstream inflammation within the intestinal epithelium [10]. A humoral response with increased levels of sIgA in mature breast milk has been shown to occur in response to an infection in the mother or infant [11]. Gopalakrishna and colleagues studied the role that sIgA in breast milk plays in the pathogenesis of NEC [12]. They determined the proportion of IgA-associated intestinal bacteria and discovered that premature infants with an exclusive formula-fed diet contained very low levels of IgA-associated intestinal bacteria. Furthermore, infants with NEC had higher levels of IgA-unbound Enterobacteriaceae when compared with healthy age-matched controls. This suggests that insufficient concentrations of IgA and decreased IgA-bound bacteria in the intestine may be causative factors of insufficient microbiome diversity and increased risk of NEC development [12]. In a neonatal mouse model of NEC, pups reared by IgA-deficient mothers showed susceptibility to intestinal disease despite receiving maternal milk, suggesting that maternal IgA can define the host-microbiota relationship and underscoring that the IgA in milk plays an important role in the susceptibility to NEC [12]. 

### 2.2. Lactoferrin

Lactoferrin is an abundant peptide in human milk and has known roles in host defense and antimicrobial properties. When lactoferrin encounters proteolysis in acidic conditions, such as in the stomach, lactoferricin is produced. Lactoferricin has both strong antibacterial and some antiviral activity with immunomodulatory capabilities [13]. In particular, human lactoferricin has a potent ability to neutralize endotoxin activity, prevent activation of mononuclear cells, and ultimately prevent the secretion of cytokines, such as interleukin (IL)-1β, IL-6, tumor necrosis factor (TNF)-α, and IL-8, that contribute to inflammation [13,14]. Togawa and colleagues demonstrated that the administration of enteral lactoferrin in rats attenuated colonic inflammation after induction of colitis [15]. Many studies have subsequently evaluated the modulatory role of lactoferrin in antimicrobial and immunological defenses in infants. A Cochrane Review of six, small, randomized control trials (RCT) that provided lactoferrin supplementation to enteral feeds found decreased late-onset bacterial and fungal sepsis in preterm infants, although the evidence was identified as low in quality [16]. A systematic review and meta-analysis reviewed nine RCTs and showed that prophylactic lactoferrin significantly reduced the incidence of late-onset sepsis and NEC (Bell’s stage II or greater) [17]. Most recently, a large randomized control trial of 2203 infants contrasted these prior findings, demonstrating that enteral supplementation with bovine lactoferrin did not reduce NEC or the incidence of infection or mortality [18]. 

### 2.3. Lysozyme

Lysozyme, as an immune-active enzyme in colostrum and breast milk, has many bactericidal effects. In synergy with lactoferrin, lysozyme can bind to lipopolysaccharide (LPS) on outer bacterial membranes, which provides lysozyme access to degrade internal proteoglycan matrices of bacterial membranes. Studies of breast milk composition have shown that premature breast milk as compared to term breast milk has higher lysozyme content [6,9], although other studies have found no difference [8]. In the gastrointestinal tract, Paneth cells within the crypts of Lieberkühn produce a variety of antimicrobial peptides, including lysozyme, which are secreted in response to enteric pathogens [19]. Of relevance to NEC, in the small intestinal biopsies of premature infants with NEC, there were decreased concentrations of Paneth cells compared to controls [20]. The role of lysozyme has been studied in a mouse model of NEC utilizing Paneth cell ablation. This model consists of 14-day-old pups treated with dithizone, a heavy metal chelator, followed by luminal infection with *Klebsiella* [21]. The pups in this experimental group developed a NEC-like injury, suggesting the significance of lysozyme and antimicrobial protection provided by Paneth cells can regulate the inflammatory response in NEC [21]. A subsequent study using an experimental murine NEC model demonstrated that Paneth cell deficiency induces a disruption in the intestinal microbiome, and in particular, the development of an Enterobacteriaceae bloom, which has been shown to precede NEC in humans [22]. These results signify the potential significance of breast milk lysozyme in protecting breast fed infants from the intestinal inflammatory insult seen in NEC.

### 2.4. Lactadherin

Lactadherin (milk fat globule-epidermal growth factor (EGF) factor VIII) is a human milk glycoprotein that contributes to apoptotic cell phagocytosis [23]. A deficiency of lactadherin has been strongly associated with inflammatory and autoimmune diseases and has been shown to maintain homeostasis of the intestinal epithelium through the migration of epithelial cells. In a model of seven-week-old mice, treatment with recombinant lactadherin resulted in protection from colitis, as demonstrated by downregulation of pro-inflammatory cytokines and improved histological scores [23]. Additionally, in a neonatal rat model of NEC-like intestinal injury, supplementation with recombinant human lactadherin attenuated the disruption of cellular tight junctions [24]. 

### 2.5. Epidermal Growth Factor

The growth factors in breast milk serve a protective role in helping to facilitate the intestinal mucosal barrier maturation. Maternal milk and colostrum contain epidermal growth factor (EGF) and are the predominant sources of intestinal EGF during the postnatal phase. The roles of EGF in the development of the intestine, as well as the response and repair of the intestine during intestinal injury or infection, have been reported [25]. EGF levels are decreased in the saliva and serum of premature infants with NEC when compared to infants without NEC. In a study of salivary EGF, infants with NEC had lower salivary EGF in the first week after birth and greater increases from week of life one to two as compared to infants without NEC, suggesting that NEC development may be attributed to overall lower EGF concentrations in the at-risk neonate [26]. EGF also has proposed effects on goblet cells and the production of mucin in the intestinal epithelium. Clark and colleagues showed that treatment with EGF resulted in an increased number of goblet cells and increased the production of mucin in the small intestine [27]. 

NEC has been associated with impaired intestinal barrier function and epithelial cell apoptosis. The in vivo treatment with enteral EGF has shown to regulate the expression of tight junction proteins, occludin and claudin-3 as well as normalize their expression at the site of NEC injury, helping to maintain the gut barrier [27]. Additionally, enteral EGF administration can increase expression of the anti-apoptotic protein, Bcl-2, and decrease levels of the pro-apoptotic protein, Bax. The role of EGF in balancing apoptosis regulators provides implications of an opportunity for future therapeutic strategies to protect the intestinal barrier from injury in NEC [28,29].

### 2.6. Heparin-Binding Epidermal Growth Factor

The developing fetus and the breast fed newborn are continually exposed to Heparin-binding epidermal growth factor (HB-EGF), which is present in both amniotic fluid and breast milk, suggesting its possible role in gastrointestinal epithelium development both in utero and during the neonatal period [30]. As a member of the EGF family, HB-EGF binds to the EGF receptor (EGFR) and has known mitogenic effects. HB-EGF is expressed in response to hypoxia, tissue damage, and oxidative stress, including in the intestine, and has a pivotal role in tissue regeneration and repair [31,32]. In seeking to evaluate the role of exogenous HB-EGF in the context of NEC, Dvorak and colleagues demonstrated that either the oral administration of HB-EGF or EGF significantly reduced NEC in a premature rat model through increased production of MUC2, a secretory mucin [33]. However, the concurrent administration of both growth factors did not confer better protection and physiologic doses of EGF provided better protection [33]. In another study, enteral administration of HB-EGF to neonatal rat pups decreased the incidence and severity of NEC and reduced intestinal permeability as demonstrated by a low serum concentration of enterally-administered fluorescein isothiocyanate-dextran [32]. The results of these studies suggest a potential role of HB-EGF in the attenuation of intestinal injury during NEC. 

### 2.7. Transforming Growth Factor-β2 

Human milk contains high concentrations of the transforming growth factor-β isoform, transforming growth factor-β2 (TGF-β2), which has immunomodulatory effects on intestinal maturation, immunoglobulin production, and a suppressive effect on T cells [34]. Breast milk with higher concentrations of TGF-β2 is associated with a higher diversity of intestinal microbial composition in the neonate, a factor that is known to lower the risk of adult immunological diseases [34]. Of note, preterm human milk has been shown to have reduced TGF-β bioactivity [35]. Maheshwari and colleagues analyzed TGF-β2 expression in premature infant intestinal tissue samples and observed lower TGF-β2 expression and bioactivity in patients with NEC as compared with controls [36]. In a murine experimental model of NEC, enterally administered recombinant TGF-β2 showed protective effects against NEC-like mucosal injury [36]. The addition of recombinant TGF-β2 to milk has been investigated as a preventative strategy to boost the anti-inflammatory properties of milk and prevent the development of NEC. However, it was discovered that in human preterm milk, TGF-β2 is sequestered by chondroitin sulfate proteoglycans, which therefore inhibits its biological activity [35]. Consequently, the digestion of human preterm milk with chondroitinase resulted in the activation of endogenous TGF-β2 and also restored the bioactivity of recombinant TGF-β2 [35]. These findings suggest chondroitinase digestion of preterm milk may be an option for preventing NEC by enhancing the anti-inflammatory properties of the milk.

### 2.8. Prebiotics and Oligosaccharides

Human milk oligosaccharides (HMOs) are complex sugars present in high abundance in breast milk. HMOs serve as prebiotics and metabolic substrates with targeted antimicrobial activity, allowing beneficial bacteria to thrive while suppressing those which are potentially harmful [37,38]. In an in vitro epithelial model of the crypt-villus axis, treatment with HMOs resulted in reduced intestinal cell proliferation, but promoted epithelial cell differentiation, indicating a potential role in intestinal maturation [39].

HMOs ingested from breast milk undergo only minimal degradation in the infant’s acidic stomach and by the pancreatic and brush border enzymes in order to reach the distal small intestine and colon [37,38]. Preclinical animal studies as well as human studies in mother–infant dyads support the contributions of HMOs in reduction of the development of NEC. In a cohort study comprised of 200 mother–infant dyads, the composition of HMOs in breast milk was analyzed [40]. One specific HMO, disialyllacto-N-tetraose (DSLNT) was identified to be present in significantly lower concentrations in those infants who developed NEC [40]. Measurement of DSLNT levels in maternal milk may therefore provide additional insight into why some breast fed infants are still at risk of NEC. 

Enteral administration with supplemental HMOs have been studied as potential therapeutics in reducing the risk of NEC [41,42]. For example, in a neonatal rat model of NEC, animals were fed with DSLNT-containing formula, which resulted in reduced severity of NEC based on pathology scores and improved survival [43]. The same study showed that galacto-oligosaccharides, an infant formula additive, similar although structurally different from HMOs, demonstrated no effect on NEC severity or survival in neonatal rats [43]. In a preterm pig model of NEC, receiving supplemental feeds with a mixture of HMOs have not shown a significant difference in NEC severity, gut microbial colonization, or intestinal permeability [42]. However, in a neonatal mouse model of NEC, enteral administration of another HMO found in breast milk, 2’fucosyllactose (HMO-2’FL), resulted in the preservation of mesenteric perfusion and restored the expression of endothelial nitric oxide synthase (eNOS), a vasodilatory molecule necessary for intestinal perfusion [44]. The results of these studies suggest key roles of HMO-2’FL and DSLNT as protective components of breast milk in the prevention of NEC development. 

### 2.9. Glutamine

Free amino acids comprise 3–5% of the total amino acids in human milk [45]. In a longitudinal analysis of breast milk from healthy mothers of term infants, glutamine and glutamic acid were among the most plentiful free amino acids in the first three months of lactation [45]. Levels of glutamine increased significantly during the first to the third month of lactation [45,46]. In addition, breast milk, which contains higher concentrations of glutamine, amongst other free amino acids, is associated with more rapid weight gain [46] and increased length [47] in the infant. Glutamine also holds a relevant role in maintaining gut barrier integrity. For example, glutamine augments the effects of growth factors and influences cell signaling pathways involved in intestinal cell proliferation and differentiation, as well as the expression of tight junctions [48,49]. Glutamine has also exhibited anti-apoptotic properties in intestinal cells, attributed to its role in the production of glutathione [48,49].

Neonates deficient in circulating amino acids such as glutamine and arginine, are associated with a higher risk of NEC development [50]. In a neonatal rat model of NEC, pups receiving exogenous administration of glutamine had reduced pathology injury scores and reduced ileal mRNA expression of the innate immune receptors, Toll-like receptor (TLR)-2 and TLR-4 [51]. As TLR-2 and TLR-4 have established roles in inducing synthesis of inflammatory mediators and increasing apoptosis in NEC [52,53,54], their reduced expression by glutamine supplementation suggests a mechanism by which it mediates protection. In a small study that evaluated the outcomes of arginine and glutamine supplementation in 25 preterm neonates of less than 34 weeks’ gestation, there were no infants that developed NEC in the glutamine group and no difference in the NEC incidence in the arginine group [55]. However, large RCTs of infants diagnosed with severe gastrointestinal disease, including NEC, spontaneous intestinal perforation, and intestinal structural anomalies, did not show a decreased risk of death or severe infections while receiving enteral glutamine [56]. Additional large RCTs evaluating glutamine supplementation in preterm infants did not show a benefit in decreasing the risk of death, intestinal disease, or long term developmental outcomes [57]. Therefore, despite the significant levels of glutamine in human breast milk, there is insufficient evidence for exogenous supplementation of glutamine as a preventative strategy for NEC at this time. 

## 3. Breast Milk and Immune Homeostasis

### 3.1. Cellular Mechanisms

There are two primary pathways for maternal cellular transfer to the infant—placental transmission and oral transmission through breastfeeding [58]. Breast milk leukocytes, including macrophages and neutrophils, survive passage through the neonatal gastrointestinal tract and translocate to blood, lymph nodes, spleen, and liver [11,58,59]. Understanding the physiological significance of the transfer of human milk cells to neonates can provide insight into the protective properties of breast milk on the infant recipient.

The progression through maturational stages of lactation involves alterations in breast milk leukocyte composition and concentration. In an analysis of CD45^+^ leukocytes in breast milk, colostrum contained the highest number of leukocytes compared with transitional milk (8–12 days postpartum) and mature milk (26–30 days postpartum) [60]. The infant’s gestational age at birth is also associated with changing concentrations of certain types of breast milk leukocytes. Colostrum contains lower levels of non-cytotoxic T cells and B lymphocytes with increased gestational age whereas mature milk of preterm mothers contains lower cytotoxic T cell and natural killer (NK) cell levels when compared to term milk [60]. In seeking to understand the impact of maternal milk leukocytes on the breastfeeding infant, Cabinian and colleagues used a murine model to examine the transport and survival of maternal breast milk leukocytes, primarily T cells, to the gastrointestinal Peyer’s patches of the suckling pup [61]. The observed transfer of cells to the Peyer’s patches implicates the role of breast milk leukocytes in neonatal intestinal development and localized immunological maturation. The overall relevance of the differences in human milk cellular content and transfer on the development of NEC requires further study.

Additionally, maternal and infant bacterial infections influenced concentrations of breast milk leukocytes and cytokines, notably macrophages and TNF-α levels [11,62]. Maternal infection can induce a significant leukocyte surge that ranges from 0.7% to 93.6% of total cells in breast milk [11]. A smaller increase in breast milk leukocytes has been observed when the breastfeeding infant develops an infection [11]. Riskin and colleagues identified that macrophages, as well as neutrophils, comprise the majority of breast milk leukocytes in mothers with a sick infant [62]. The increase in breast milk leukocytes in response to an inflammatory process in the mother/infant dyad suggests a dynamic interaction between maternal and infant immune systems and further supports the benefits of breast milk.

### 3.2. Cytokines

Preterm infants, when compared with their term counterparts, exhibit immune immaturity, which includes lower production of cytokines and other immunological proteins during challenge with an inflammatory insult [59]. The presence of cytokines in breast milk provides passive protection and immune modulation in the infant recipient and results in absorption into the systemic circulation. In particular, these cytokines include IL-1, IL-2, IL-6, IL-8, IL-10, interferon (IFN)-γ, and TNF-α **(**Table 1). Breast milk produced by mothers of full-term infants contains high levels of IL-2, IL-8, and IL-10, with levels decreasing drastically by day 21 of lactation. In contrast, mothers of preterm infants have significantly lower levels of cytokines in the colostrum when compared to mothers of full-term infants [63]. 

High levels IL-1RA, an IL-1 receptor, have been detected in breast milk [64]. IL-1β is a member of the IL-1 family and is known to induce an endogenous innate inflammatory response in enterocytes, upregulate expression of pro-inflammatory IL-8, and stimulate the nuclear factor kappa beta (NF-kB) pathway. However, human milk has demonstrated the ability to attenuate the IL-1β-dependent activation of IL-8 [64]. The protective effects of breast milk on suppressing this NF-kB-mediated pro-inflammatory immune response has been shown in intestinal epithelial cells both in vitro and in vivo, providing evidence of a mechanism mediating protection against NEC development [53,64]. 

Neonates have a known deficiency in the production of IL-2, which is a necessary cytokine in the recruitment of T cells required to produce an antigen-specific immune response [65,66]. Human milk, therefore, provides an ideal source of IL-2 for the newborn. Levels of aqueous IL-2 in human milk are of highest concentration in colostrum with reduced levels in later stages of lactation [63,65]. The presence of IL-2 in breast milk, which is absorbed by the gastrointestinal tract of the infant, may enter the systemic circulation to influence the maturing immune system. 

IL-6 is a pro-inflammatory cytokine in the acute phase of the inflammatory response [59]. Multiple studies have observed high levels of IL-6 in colostrum [63,67,68]. An early analysis of IL-6 in breast milk showed that the presence of an anti-IL-6 antibody in colostrum caused decreased production of IgA by mononuclear leukocytes, suggesting a relationship between IL-6 and IgA production in breast milk [67]. Ustundag and colleagues noted higher levels of breast milk IL-6 at two weeks postpartum in mothers of term infants when compared to milk from mothers of preterm infants [63]. The prevalence of IL-6 in breast milk with uptake by the infant recipient may have a significant biologic role in neonatal immune homeostasis.

IL-8 expression by macrophages, endothelial cells, and epithelial cells provides chemotactic activity for a neutrophil-dependent response to acute inflammation, such as in sepsis and NEC. A decline in IL-8 levels in breast milk occurs with the advancement in lactational stage [69,70]. Although one study found no difference in breast milk IL-8 expression in mothers of infants of different gestational ages [63], others have identified higher IL-8 levels in the breast milk of mothers of preterm infants [69]. Maheshwari and colleagues showed that fetal and adult human intestinal cells treated with recombinant IL-8 in vitro had increased cell proliferation and differentiation [69]. Additionally, intestinal cells exposed to injury in vitro demonstrated increased viability when treated with recombinant IL-8 [69]. Thus, the dynamic effects of IL-8 on the developing intestine suggests its physiologic role in intestinal development as a component of human breast milk.

The anti-inflammatory properties of IL-10 attenuate the immune response to an infection and maintain tissue homeostasis by inhibition of the activity of T_h_1 effector cells, NK cells, and macrophages [71,72]. IL-10 can inhibit the production of inflammatory IL-1, IL-6, IL-8, and TNF-α [72,73]. One study found IL-10 in the aqueous and non-aqueous phases of human milk, with concentrations found to be highest within the first 24 hours of lactation [72]. IL-10 in breast milk affects the infant by attracting CD8^+^ T lymphocytes [72,74] and promoting immunoglobulin synthesis by B cells [72,75].

IFN-γ is a pro-inflammatory cytokine found in human milk in low concentrations with decreasing levels in the months following birth [76]. IFN-γ is secreted by activated T cells and NK cells and enhances intestinal macrophage activation [76]. IFN-γ is involved in the signaling pathways that increase intestinal epithelial barrier permeability [77] and is also detected in higher concentrations in the ileum of patients with NEC [78]. As infants have a reduced ability to produce IFN-γ due to an immature immune system [79], breast milk may provide the infant with IFN-γ and other pro-inflammatory cytokines needed to produce a host defense response against inflammation or infection.

Neonates are deficient in the production of the pro-inflammatory cytokine TNF-α and its receptors, TNF-α receptor I and II, increasing susceptibility to infection due to immune cell dysregulation. TNF-α is produced by a variety of immune cell types, including granulocytes and CD4^+^ lymphocytes. TNF-α, as an endogenous pyrogen, contributes to systemic inflammation and immune cell regulation. One study quantified the amount of detectable TNF-α in breast milk and colostrum and identified the majority of TNF-α to be in association with its soluble receptor [80]. The low amount of unbound TNF-α in breast milk was theorized to decrease TNF-α pro-inflammatory bioactivity [80]. Two additional studies have identified a significantly decreased amount of TNF-α in colostrum of mothers who delivered very preterm (less than 30 weeks’ gestation) when compared to term and preterm groups, suggesting one reason why preterm infants have increased susceptibility to infection and impaired immunity [63,81].

Overall, the evidence suggests that infants, particularly those born preterm, have insufficient ability to mount an adequate immunological defense due to reduced production of a variety of cytokines. Breast milk can therefore supplement infants with maternal cytokines that may provide immune benefits in the protection against neonatal inflammatory diseases such as NEC.

### 3.3. Nitric Oxide

Nitric oxide (NO) is a soluble molecule produced by isoforms of NO synthase (NOS) and serves as a potent vasodilator and neurotransmitter at low, physiologic levels [82,83]. Infants derive NO from dietary sodium nitrite, which is then converted to NO within the gastrointestinal tract by commensal microbes [82,83]. NO is present in breast milk, as shown in an analysis of healthy lactating mothers evaluating the concentration of breast milk nitric oxide concentration on postpartum days one through five [84]. Exclusively breastfeeding mothers had significantly higher nitric oxide concentrations in their milk, compared with milk expressed from mothers who decided to exclusively formula-feed their infant [84]. It was theorized that infant suckling activates NOS within the mammary gland with subsequent secretion of NO into breast milk, which then confers protection to the intestine of the infant through regulation of intestinal blood flow and maintenance of vascular tone [84]. 

The upregulation of inducible NOS (iNOS) in response to the release of cytokines and growth factors has been implicated in NEC pathogenesis [82,85]. During inflammation, high levels of NO, and its derivative peroxynitrite, contribute to epithelial damage and the disruption of the integrity of the intestinal barrier [82]. To study abnormal NOS signaling in NEC, Yazji and colleagues used a murine model of NEC and selectively deleted endothelial TLR-4 expression, which subsequently resulted in impaired microvascular intestinal perfusion, increased severity of NEC, and reduced endothelial NOS (eNOS) expression [86]. Additionally, as compared with commercial formula, breast milk was identified to have higher levels of sodium nitrate, which serves as a precursor for nitrite and nitric oxide. Enteral administration of exogenous sodium nitrate was associated with decreased severity of NEC and improved intestinal perfusion [86]. Overall, these results suggest the protective role of breast milk in augmenting physiologic nitrate-nitrite-NO signaling to improve intestinal vascular perfusion and protect against intestinal barrier disruption in NEC. 

## 4. Summary

Human breast milk contains a dynamic diversity of bioactive components needed for infant growth, immune homeostasis, and intestinal maturation. The composition of human milk varies with the stage of lactation, gestational age of the infant, the health of the mother/infant dyad, and the nutritional status of the mother. The dietary intake of the breastfeeding mother has been shown to influence the variability of human milk concentrations of fat-soluble and water-soluble vitamins and other nutrients. These nutrients, including immunoglobulins, growth factors, cytokines, and immune cells, have been demonstrated to transfer from the mother to the neonate through breast milk [87,88]. The ability of these components to regulate intestinal cell proliferation and differentiation as well as influence gut microbial colonization emphasizes the protective role of breast milk in infant metabolism and neurodevelopment, intestinal microbial homeostasis, and protection against NEC [87,88]. The growing field of research studying the outcomes related to breastfeeding reinforces the immunological value of breast milk on infant nutrition and protection from inflammatory disorders such as NEC.

## Figures and Tables

**Table 1:** Cytokines present in human breast milk and physiologic relevance to the infant.

Cytokine	Composition in Human Milk and Significance	References
Interleukin (IL)-1	- Human milk IL-1β attenuates the activation of pro-inflammatory IL-8 and suppresses pro-inflammatory responses of nuclear factor kappa beta (NF-kB) signaling.	[53,64]
IL-2	- Highest in concentration in colostrum and reduced in later stages of lactation. - Recruits T cells to stimulate an antigen-specific immune response.	[63,65,66]
IL-6	- Detected in higher levels in term breast milk. - Pro-inflammatory properties and is present in the acute phase of infection. - Colostrum may contain anti-IL-6 antibodies that cause decreased immunoglobulin A (IgA) production by breast milk leukocytes.	[63,67,68]
IL-8	- Decreased levels of detection in later stages of lactation. - Provides chemotactic response of neutrophils. - Recombinant IL-8 may improve the viability of intestinal cells when exposed to injury.	[63,69,70]
IL-10	- Maintains anti-inflammatory mechanisms involving limiting the T_h_1 response, inhibiting production of inflammatory cytokines, and promoting immunoglobulin synthesis.	[71,72,73,74,75]
IFN-γ	- Detected in decreasing levels with later stages of lactation. - Increases activation of intestinal macrophages and is present in higher concentrations in the ileum of infants with necrotizing enterocolitis (NEC). - Pro-inflammatory mechanism of action may provide an infant with defense against inflammation and infection.	[76,77,78,79]
TNF-α	- Detected in decreased levels in colostrum of preterm milk. - Present in breast milk in association with its soluble receptor, reducing its pro-inflammatory activity.	[63,80,81]
